# Fine-Scale Spatial Variability of Greenhouse Gas Emissions From a Subantarctic Peatland Bog

**DOI:** 10.1021/acs.est.3c10746

**Published:** 2024-04-16

**Authors:** Brenda Riquelme del Río, Armando Sepulveda-Jauregui, Julio A. Salas-Rabaza, Roy Mackenzie, Frederic Thalasso

**Affiliations:** † Cape Horn International Center, Universidad de Magallanes, Teniente Muñoz 166, Puerto Williams 6350000,Chile; ‡ Millennium Institute Biodiversity of Antarctic and Subantarctic Ecosystems (BASE), Las Palmeras, 3425, Santiago 7800003, Chile; § Environmental Biogeochemistry Laboratory, Centro de Investigación Gaia Antártica (CIGA), Universidad de Magallanes, Av. Bulnes 01855, Punta Arenas 6210427, Chile; ∥ Ecosystem Processes, Plankton and Microbial Ecology, IGB Leibniz-Institute of Freshwater Ecology and Inland Fisheries, Zur alten Fischerhütte 2, Stechlin 16775, Germany; ⊥ Departamento de Biotecnología y Bioingeniería, Centro de Investigación y de Estudios Avanzados del Instituto Politécnico Nacional (Cinvestav), Av. IPN 2508, Mexico City 07360, Mexico

**Keywords:** Southern Chile, methane, carbon dioxide, heterogeneities, temporal variations, numerical model

## Abstract

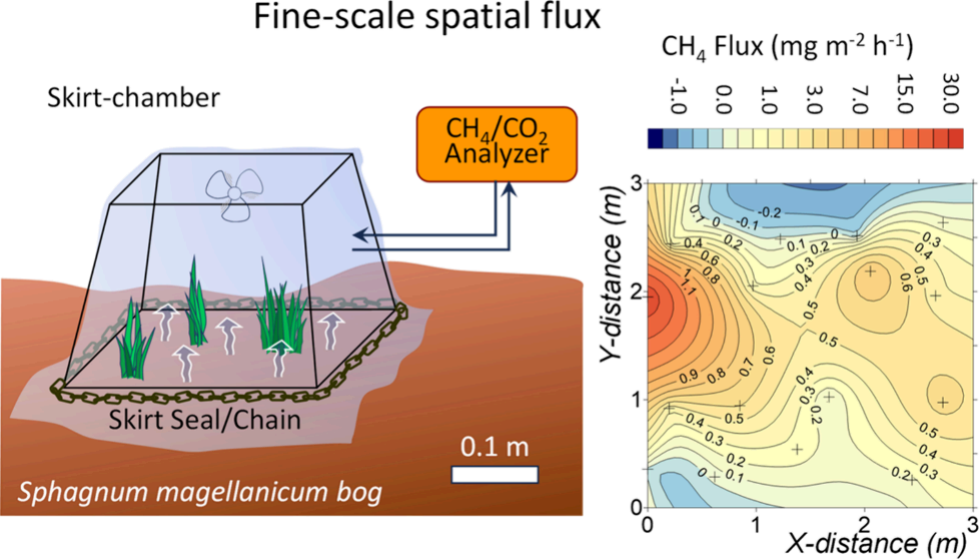

Peatlands are recognized as crucial greenhouse gas sources and sinks and have been extensively studied. Their emissions exhibit high spatial heterogeneity when measured on site using flux chambers. However, the mechanism by which this spatial variability behaves on a very fine scale remains unclear. This study investigates the fine-scale spatial variability of greenhouse gas emissions from a subantarctic *Sphagnum* peatland bog. Using a recently developed skirt chamber, methane emissions and ecosystem respiration (as carbon dioxide) were measured at a submeter scale resolution, at five specific 3 × 3 m plots, which were examined across the site throughout a single campaign during the Austral summer season. The results indicated that methane fluxes were significantly less homogeneously distributed compared with ecosystem respiration. Furthermore, we established that the spatial variation scale, i.e., the minimum spatial domain over which notable changes in methane emissions and ecosystem respiration occur, was <0.56 m^2^. Factors such as ground height relative to the water table and vegetation coverage were analyzed. It was observed that *Tetroncium magellanicum* exhibited a notable correlation with higher methane fluxes, likely because of the aerenchymatous nature of this species, facilitating gas transport. This study advances understanding of gas exchange patterns in peatlands but also emphasizes the need for further efforts for characterizing spatial dynamics at a very fine scale for precise greenhouse gas budget assessment.

## Abstract 2

Peatland bogs are significant greenhouse gas sources/sinks that exhibit fine-scale variability, which is critical for quantifying the precise greenhouse gas budget.

## 1 Introduction

Peatlands play an important role in the global carbon cycle and are recognized as the largest carbon reservoir in the biosphere.^1^ These ecosystems store a substantial amount of carbon, ∼644 gigatons (Gt), across 399 million hectares.^2,3^ In Chile, peatlands store approximately five times more carbon than the entire aboveground biomass of forests.^4^ Consequently, peatlands have emerged as potential nature-based solutions for addressing global warming.^5,6^ Peatlands are estimated to function as carbon sinks globally, sequestering ∼0.1 Gt of carbon annually;^7^ however, they are also significant emitters of methane (CH_4_) into the atmosphere.^8^ Their behavior as either greenhouse gas (GHG) sinks or net sources varies across different temporal and spatial scales and is influenced by various factors, including but not limited to climatic conditions, hydrology, and anthropogenic impacts.

Previous studies have investigated the GHG budget of peatlands, primarily focusing on carbon dioxide (CO_2_) and CH_4_. Two common methodologies are employed in these studies. The first method employs aboveground techniques, specifically eddy covariance (EC) methods,^9^ which continuously monitor emissions and provide high temporal resolution. However, their drawback lies in averaging data over the entire ecosystem footprint, thereby overlooking the fine-scale spatial variability. The second approach uses ground-based methods, employing chambers positioned on the surface of the terrain to measure GHG fluxes at precise locations, offering better spatial resolution than aboveground techniques. However, standard chambers have several inherent limitations. First, chambers must maintain effective seals to prevent gaseous exchange with the atmosphere, which is achieved by installing collars into the ground by cutting vegetation and roots up to 30 cm deep, potentially impacting the integrity of the soil and its underlying processes.^10,11^ Thus, a common practice is to observe a waiting period after installation before flux measurements are begun to reduce ground perturbation. Second, the placement of collars for sealing purposes might orientate the selection of the locations toward terrains that are compatible with the collar design and potentially exclude considerably irregular topographies. Third, while automatic chambers provide enhanced temporal resolution, they often come at the expense of spatial resolution. This limitation is attributed to the cost of the chambers, which restricts their numbers when deployed simultaneously.^12^

Thus, current methods favor temporal resolution, and previous studies on the spatiotemporal variation of GHG emissions in peatlands have focused on high temporal resolution. However, their spatial resolution was typically limited to a relatively small number of locations—usually ranging from <10 to 20,^13−18^ located at distances of 3–50 m from each other, and thus with an indicative spatial resolution in the range of tens of meters. This approach has sometimes diverted attention from the inherent spatial variability of GHG emissions, which respond significantly to several key factors varying across spatial scales, such as the water table levels,^19,20^ local topography,^21^ and the vegetational cover, among others. These factors present considerable spatial variability at the peatland surface,^21−23^ occurring at a small scale, typically within the meter range, and has to be further constrained to better capture the nuanced dynamics of GHG emissions in peatlands.

The “skirt chamber” introduced by Thalasso et al.^24^ is a novel tool for detecting fine-scale GHG emissions in peatlands. Its noninvasiveness, portability, and rapid deployment render it potentially efficient for studying the spatial variations of GHG emissions at a high resolution, allowing deployments at very short distances, approximately the size of the chamber (≤0.3 m), i.e., in the submeter range. This study investigated the fine-scale dynamics of CH_4_ emissions and CO_2_ respiration in a peatland. Our study was conducted in a peatland dominated by *Sphagnum magellanicum* located on the Navarino Island in the subantarctic ecoregion of Chile (Lat. 54.9° S) where peatlands comprise the dominant ecosystem and yet are understudied. In this context, our aim extended to the study of Chilean Patagonian peatlands that span an area of 3.1 million hectares and hold ∼4800 million tons of carbon, equivalent to 4.7 times the carbon content found in Chile’s aboveground biomass of forests.^4^

## 2 Material and Methods

### 2.1 Skirt Chamber Method

The details of the skirt chamber and its associated method have been previously documented by Thalasso et al.^24^ In summary, the chamber design comprises an open frame constructed with sparsely interwoven steel wires, serving to support a transparent plastic film that defines the chamber’s volume (Figure S1). This film is securely attached to the chamber, which is positioned downward when it is installed on the ground. During installation, the plastic film is extended around the chamber and on the ground, and a steel chain is placed above the film and around the base of the chamber to ensure proper contact with the ground. This configuration creates a fixed-volume chamber that remains open at the bottom and is in direct contact with the ground. A fan is placed inside to homogenize the air content within the chamber. Inlet and outlet ports, positioned on opposite sides of the frame, are connected in a recirculation mode to an infrared laser spectroscopy greenhouse analyzer (UGGA, model 915–0011–1000, Los Gatos Inc., ABB, USA), which measures CH_4_ and CO_2_ concentrations at 1 Hz together and continuously.

The mass balance of the chamber gas phase is expressed as follows.
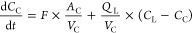
1

In eq 1, the variables are defined as follows: *C*_C_ represents the gas concentration (CH_4_ or CO_2_) inside the chamber (mg m^–3^); *F* denotes the flux (mg m^–2^ h^–1^); *A*_C_ represents the area of the chamber in contact with the ground (m^2^); *V*_C_ represents the chamber volume (m^3^); *Q*_L_ indicates the flow rate of gas exchange between the chamber and the exterior due to the imperfect seal between the chamber and the ground, i.e., leaks (m^3^ h^–1^); and *C*_L_ indicates the gas concentration at ground level outside the chamber. Additionally, *Q*_L_/*V*_C_ is the dilution rate caused by the gas exchange between the chamber and the environment due to leaks, which is the inverse of the mean residence time of the gas within the chamber (θ_C_).

At equilibrium, when the chamber reaches a steady state, *i.e*., concentration not fluctuating over time, d*C*_C_/*d*t equals zero and the chamber’s gas concentration can be considered the constant *C*_B_ (baseline concentration). Under these conditions, eq 1 becomes
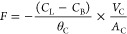
2

As *V*_C_ and *A*_C_ are known, *F* can be determined during chamber deployment from the measurement of *C*_L_, *C*_B_, and θ_C_, as detailed in Thalasso et al.^24^ and according to a four-step protocol, described in detail in Section S2 of the Supporting Information. Step 1: the ground air concentrations (*C*_L_) of CH_4_ (*C*_L,CH4_) and CO_2_ (*C*_L,CO2_) are measured for 5 min just above the vegetation cover (where the chamber is placed); step 2: the chamber is positioned, and the gas concentration inside it is measured. Once a steady state is reached, *C*_B_ of CH_4_ (*C*_B,CH4_) is measured over 5 min and used to determine the CH_4_ flux (*F*_CH4_); step 3: a pulse of about 1 mL of standard CH_4_ (99.99%, Linde, Chile) is injected with a plastic syringe through a septum connected to the waste line of the UGGA (returning to the chamber) and used to determine θ_C_. This step is maintained for 5–7 min until a stable CH_4_ concentration is observed; step 4: a dark fabric knitted to match the dimensions of the chamber, is positioned over it for 5 min to ensure complete darkness and to measure the CO_2_ flux in the absence of light (respiration; *R*_CO2_). Notably, the skirt chamber captures and measures the total flux, encompassing both ebullition and diffusive emission modes reaching the chamber. This approach is briefly discussed in Section S3.

### 2.2 Study Site, Campaign, and Flux Measurements

The selected research site is an ombrotrophic peatland (bog) characterized by the prevalence of *Sphagnum magellanicum* moss. This site is located at coordinates −54.940, – 67.644, and ∼2 km west of Puerto Williams, along the northern coast of Navarino Island, Southern Chile, and it sits at an altitude of about 20 m above sea level. This peatland encompasses an area of 46,000 m^2^ and is informally referred to as the “Omora peatland” as it is located within the Omora Ethnobotanical Park,^25^ established in 2000. It is alternatively referred to as “Caleta Róbalo”.^26^ This peatland has never been exploited for any purpose, and access is restricted to researchers who traverse the area using wooden boardwalks to reach the primary study site where an EC tower is installed. When accessing other areas, researchers utilize snowshoes. Consequently, the study site can be considered mostly undisturbed. During the present campaign, specific measurement locations were marked before data collection in addition to snowshoes to minimize any potential disruption to the peatland’s surface structure caused by personnel. The terrain exhibits a hummocky topography interspersed with irregular patches of vascular plants, lichens, and bare peat without live *Sphagnum* cover. Peat depth ranges from 3 to 10 m, with an average depth of 8 ± 1 m, where our experiments were conducted. The peatland is nonsubmerged, with the water table depth typically ranging from 0.1 to 0.6 m. The fieldwork was conducted during March 10–30, 2022, aligning with the conclusion of the summer season and the end of the growing season. Measurements were taken during the day, between 10:00 and 17:00, i.e., at least 2.5 h after sunrise and before sunset. Our campaign’s average daytime air temperature ranged from 2.8 to 11.9 °C (mean 7.07 °C), with a mean precipitation of 2.75 mm d^–1^.

### 2.3 Sampling Protocol

In our peatland study, five 3 × 3 m areas were chosen, each considered a distinct measurement plot. Within each plot, a grid of nine smaller squares measuring 1 m^2^ was established. CH_4_ and CO_2_ fluxes were measured near the intersecting points of this grid, resulting in 16 measurement locations within each plot. This systematic approach provided a representative assessment of the flux distribution across each measurement plot. One plot (plot 1) was measured on three different dates, i.e., 2 and 12 days after the first measurement, to account for temporal variability. The four other plots were measured once. Following each flux measurement, plastic rulers were positioned along the chamber’s base before removing the chamber to restrict the area it had covered. A photograph was then taken to document and outline the extent of plant species’ coverage. Subsequently, these scaled photographs were analyzed using the Fiji software.^27^ The software’s freehand selection tool was employed to determine the percentage of coverage for each specific plant species or group of species. The dominant species at each location was chosen based on the species’ relative abundance, i.e., when a species’ abundance is >50% or when a species has the highest abundance value among all species in each location. Furthermore, to quantify diversity, we used the Shannon Diversity Index (SDI).^28^

After measuring all fluxes within each plot (to minimize disturbance), we determined the exact position of each measurement spot through triangulation to define *X* and *Y* distances relative to the inferior-left corner of the quadrant (0, 0). The depth of the water table (*H*) was manually measured near each plot using a groundwater monitoring well consisting of a 2 in. perforated plastic tubing installed 2 days before measurements. We assessed the height of each measurement point relative to *H* using a water-level hose.

### 2.4 Data Treatment and Statistical Analysis

In all instances, the collected data were used to generate maps through interpolation using Surfer 11.0 software (Golden Software, USA). The selection of the most appropriate interpolation method, chosen from options including inverse distance to power, Kriging, minimum curvature, natural neighbor, nearest neighbor, radial basis function, and modified Shepard’s method—all of which are integrated into the Surfer software—was determined based on two primary criteria: the mean absolute error (MAE) and the mean bias error (MBE^29^). Kriging demonstrated the lowest overall MAE and MBE among the interpolation methods tested although not uniformly across all parameters and locations. However, to maintain consistency in our data treatment and interpretation, all field measurement data were interpolated by using the same Kriging method with a 50 × 50 grid (i.e., 2500 interpolation nodes). The data matrices interpolated by Surfer were used for statistical purposes, comparison, and correlation among various parameters.

We analyzed the spatial distribution of CH_4_ and CO_2_ fluxes at each plot using a modified version of the numerical homogeneity model (NHM^30^). Briefly, the model establishes several numerical attributes of the spatial distribution for any parameter measured in a given area. With this purpose, the set of flux measurements (*F* = *F*_*1*_, *F*_2_, ... *F*_i_, ... *F*_*n*_) is associated with a set of corresponding areas (*A* = *A*_1_, *A*_2_, ... *A*_*i*_, ... *A*_*n*_). Next, vector *F* is reordered so that *F*_1_ ≥ *F*_2_ ≥ ... ≥ *F*_*n*_, keeping the corresponding *A*_*i*_ for each *F*_*i*_. Furthermore, the flux data *F*_*i*_ is multiplied by the corresponding *A*_*i*_ to obtain a quantity of CH_4_ or CO_2_ emitted per unit of time (*M* = *M*_1_, *M*_2_, ... *M*_i_,... *M*_*n*_). Finally, a cumulative normalized parameter *M*′_*j*_ (eq 3) is established ranging from 0 to 1, which is coupled to a cumulative normalized *A*′ function (eq 4).
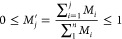
3
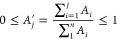
4

The spatial distribution of fluxes in each quadrant of the peatland is then obtained by plotting the pair (*M*′_*j*_*, A*′_*j*_), exemplified in Figure S2, which allows for the determination of several numerical parameters describing the heterogeneity and other spatial dispersion attributes. First, heterogeneity is quantified using *ht* (%; eq 5), ranging from 0% for an ideally homogeneous distribution to 100% for a completely nonhomogeneous distribution.
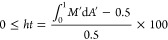
5

To understand this parameter better, the area under the curve formed is considered by plotting the pairs (*M*′_*j*_, *A*′_*j*_) (Figure S2). From this total area, a value of 0.5 is subtracted, representing the area of a perfectly homogeneous distribution (depicted as the purple area in Figure S2). This subtraction effectively emphasizes the deviation from homogeneity, highlighted by the remaining area of the curve (depicted as the orange area in Figure S2). Finally, the heterogeneity area is divided by 0.5 and then multiplied by 100 to scale it to a range of 0–100%. Notably, in the presence of significant negative fluxes, *ht* may slightly exceed 100%, as exemplified by plot 4, which is discussed later in this paper.

In addition, the NHM enables the determination of three additional numerical parameters: (i) the percentage of the area where negative fluxes (*F*_CH4_ or *R*_CO2_) are observed, indicating the capture of CH_4_ or CO_2_ from the atmosphere and reflected by *A*^<0^ (%). This area is identified as the section of the *M*′_*j*_ curve with a negative slope (Figure S2). (ii) The percentage of the area where emissions are more than five times the mean emission (hotspots), denoted as *A*^5×^ (%), which is easily established from the intersection of the *M*′_*j*_ curve with a straight line and a slope of 5 (Figure S2). (iii) The proportion of the study area that contributes to a given percentage of the total emissions, denoted by the superscript, e.g., *A*^90^ (%), represents the fraction of the area responsible for 90% of the total emissions. This corresponds to *A*′_*j*_ at which *M*′_*j*_ equals 0.9 (Figure S2). Each parameter can have a subscript added to describe the associated parameters, e.g., *ht*_FCH4_, *A*_RCO2_^90^, or *A*_FCH4_^5×^.

The NHM can be applied to measured or interpolated data, with the latter being the chosen option in the present study. Moreover, the model is not limited to fluxes data but can be applied to any quantitative or nonquantitative numerical parameter, although in that case, *M*′*j* no longer necessarily signifies a mass emitted per unit of time but a magnitude associated with the parameter under consideration. For further information regarding the NHM, please refer to previous studies that used a similar approach in aquatic ecosystems^30,31^ and landfills.^32,33^

To estimate the temporal and spatial variability of flux measurements at different times and locations, our main indicator was the mean coefficient of variation (CV), calculated as the ratio of the standard deviation to the mean. We first assessed the normality of the data using the Shapiro–Wilk test to ensure the validity of comparisons among parameters across locations and times. In cases in which the data were not normally distributed, a log_10_ transformation was applied. Notably, owing to the presence of low and negative *F*_CH4_, we utilized the transformation function (sign(*F*_CH4_) × log(abs(*F*_CH4_) + 1)) for statistical tests and graphical representations.

Significant differences among variables were determined using independent sample *t* tests, with a significance threshold of a *p* value of <0.05 unless otherwise specified. We computed correlation coefficients among parameters using Pearson’s and Spearman’s rank correlation methods.^34^ Additionally, we assessed the impact of sample size (ranging 2–100) on the standard error of the mean (SEM) for *F*_CH4_ and *R*_CO2_ through bootstrapping. This involved 1000 resampling of our complete data set in each case, i.e., drawing samples with replacement.

All statistical analyses were conducted using OriginPro (Version 2016, Northampton, USA), except when investigating the influence of species dominance on *F*_CH4_. The latter was analyzed using covariance test (ANCOVA) through JASP version 17.03.^35^ Additionally, we used Tukey’s honestly significant difference test for specific group comparisons to determine statistical significance.

## 3 Results and Discussion

### 3.1 CH_4_ Emissions

**Figure 1:**
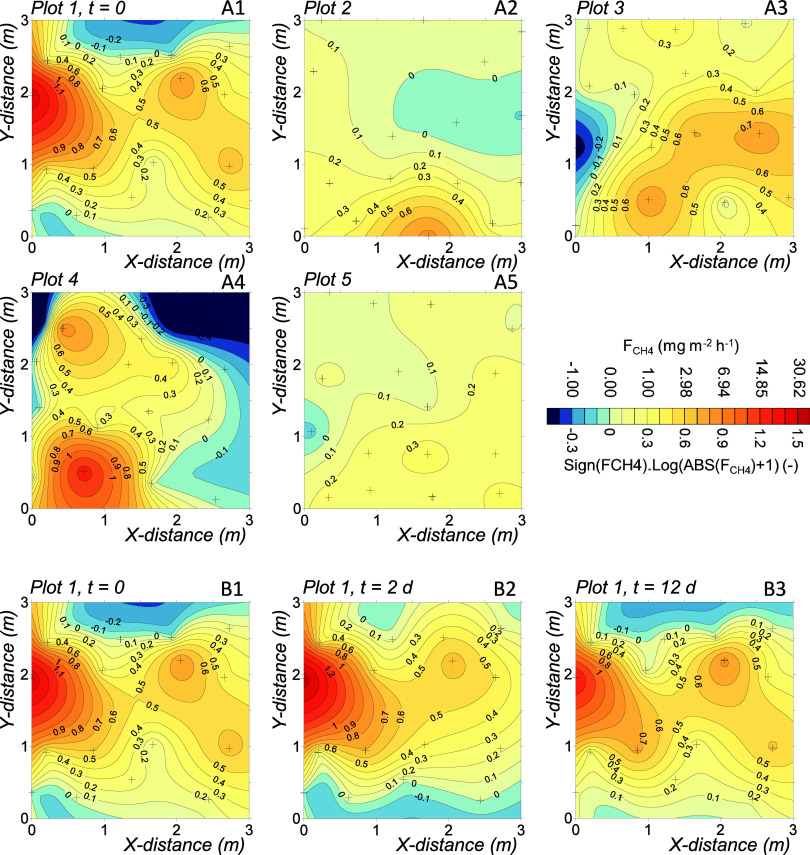
CH_4_ emission heatmaps (*F*_CH4_) over five distinct 3 × 3 plots (A1–A5) and over three dates (B1–B3; days 0, 2, and 12) on the same plot as A1. For improved visibility and interpretation, the color scale is derived from the logarithmic value of *F*_CH4_ (sign(*F*_CH4_) × log(abs(*F*_CH4_) + 1)). A linear scale is also provided. Blue areas depict negative values of *F*_CH4_. Crosses indicate the measurement coordinates.

CH_4_ emissions varied considerably over 80 locations measured (5 plots ×16 measurements), with *F*_CH4_ ranging from −4.23 to 35.26 mg m^–2^ h^–1^ and a mean of 1.82 ± 5.01 mg m^–2^ h^–1^ (one standard deviation), along with a CV of 275%. This range is consistent with values reported in previous measurements conducted in peatlands from Southern Patagonian, which ranged between −0.03 and 17.30 mg m^–2^ h^–1^.^36,37^ Five heatmaps were generated for the five plots (Figure 1A; A1–A5), and three heatmaps were elaborated for plot 1, corresponding to three distinct dates, to account for temporal variations (Figure 1B; B1–B3).

Negative *F*_CH4_ values were consistently found at each plot (depicted as blue areas in Figure 1), covering 2.4–32.7% of the area of each plot (*A*_FCH4_^<0^; Table 1). To support this observation, it is important to emphasize that negative *F*_CH4_ values were experimentally measured and thus precisely represent the data rather than being a potential artifact of data interpolation. Negative *F*_CH4_ values match the literature and possibly result from the methanotrophic activity often found in the upper layer of the peatland.^36,38,39^ Despite the observed capture of CH_4_, the net mean *F*_CH4_ was consistently positive among each plot, ranging from 0.48 to 2.37 mg m^–2^ h^–1^ (Table 1).

**Table 1:** Mean Indicators of CH_4_ Emissions and Spatiotemporal Attributes^a^

	mean *F*_CH4_ (mg m^–2^ h^–1^)	CV *F*_CH4_ (%)	*A*_FCH4_^5×^ (%)	*A*_FCH4_^<0^ (%)	*A*_FCH4_^90^ (%)	*ht*_FCH4_ (%)
plot 1 (*t* = 0)	2.43	157.2	4.4	14.0	45.6	66.0
plot 1 (*t* = 2 d)	2.66	173.9	4.7	16.2	42.7	69.5
plot 1 (*t* = 12 d)	2.01	128.6	2.5	12.6	48.4	60.4
mean plot 1	2.37	153.2	3.9	14.3	45.6	65.3
plot 2	0.55	168.0	4.8	18.2	36.8	73.5
plot 3	1.58	85.1	0.0	6.4	57.3	47.9
plot 4	1.59	208.5	6.8	32.7	21.9	104.4
plot 5	0.48	62.3	0.0	2.4	68.2	35.6
mean 5 plots	1.31	135.4	3.1	14.8	46.0	65.3
CV 5 plots (%)	60.7	44.6	97.5	79.9	39.0	40.3

a CV, coefficient of variation; *A*_FCH4_^5×^, hotspot area; *A*_FCH4_^<0^, negative area; *A*_FCH4_^90^, area that contribute 90% of total emissions; *ht*_FCH4_, heterogeneity parameter (see text for details).

**Figure 2:**
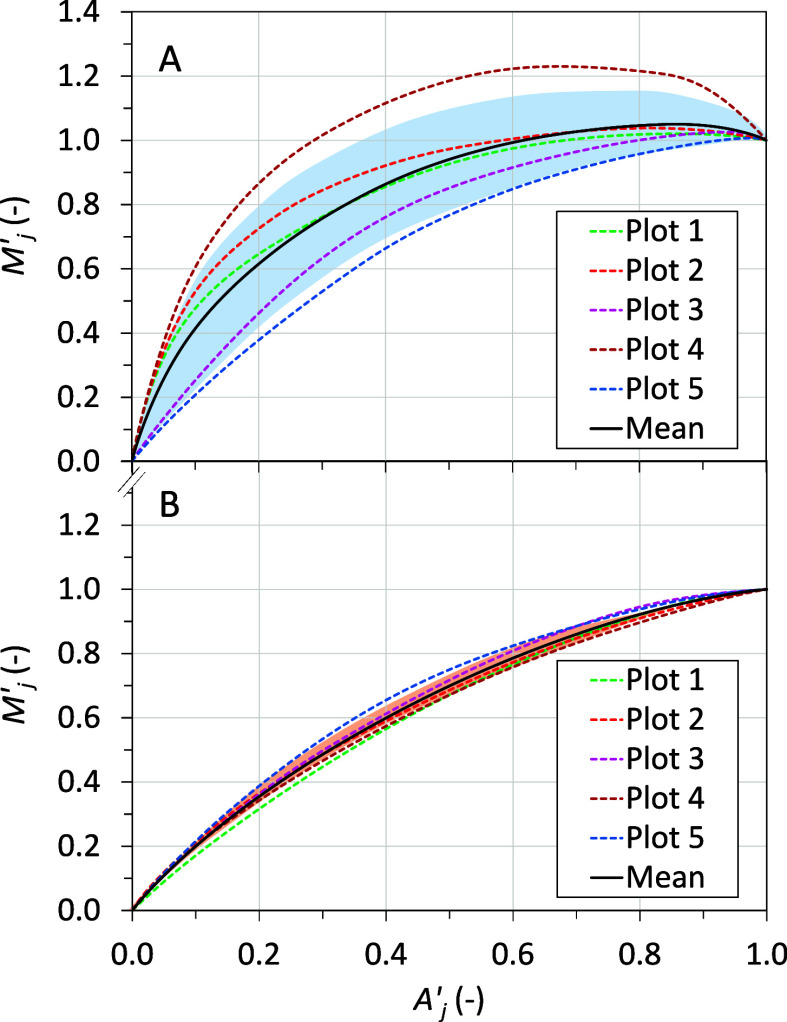
Graphical representation of the numerical homogeneity model (NHM) for *F*_CH4_ (A) and *R*_CO2_ (B) at five plots, illustrating the pair formed by the normalized cumulated and ordered mass of CH_4_/CO_2_ emitted by the plot (*M*′_FCH4_^5×^, hotspot area; *A*_FCH4_^<0^, negative area; *A*_FCH4_^90^, area that contribute 90% of total emissions; *ht*_FCH4_, heterogeneity parameter (see text for details)) and the corresponding normalized cumulated area (*A*′; eqs 3 and 4); refer to the text for additional details. Dashed lines represent plot-specific results, the solid line depicts the mean behavior, and the shaded area in blue/pink shows the ±1 standard deviation range.

Regarding fine-scale spatial variability, we observed relatively large gradients. Two examples included a 25-fold increase in *F*_CH4_ over a distance of 0.53 m in plot 1 and a gradient ranging from −4.23 to 6.34 mg m^–2^ h^–1^ over 0.35 m in plot 5. The attributes of spatial variability at a short scale are depicted in Table 1, and a graphical representation of the NHM is presented in Figure 2. The first and most obvious parameter indicating considerable spatial variation was the CV of *F*_CH4_ within each plot, which ranged from 62.3 to 208.5% with a mean of 135.4 ± 60.4%. Among the NHM parameters, *ht*_FCH4_ ranged from 36 to 104% with a mean of 65 ± 26%, indicating a significant deviation from a homogeneous distribution (*ht*_FCH4_ = 0%). Similarly, *A*^90^ ranged from 21.9 to 68.2%, implying that in the most extreme case, 90% of the total net emission of a plot was coming from approximately one-fifth of its area. Another notable feature of *F*_CH4_ was that in three plots (plots 1, 2, and 4), we observed hotspots, defined as *F*_CH4_ values at least five times higher than the mean value (Table 1). Overall, these hotspots represented 0.0–6.8% of the area of the plots, with a mean of 3.1% (*A*_FCH4_^5×^, Table1).

Herein, it is crucial to ascertain whether the observed variability within each plot could be attributed to temporal or spatial factors. Considering that measurements were not conducted simultaneously across the 16 locations within each plot, the possibility of significant temporal variability, which could be mistaken for spatial variability, could not be discounted. Thus, we conducted three complete flux measurements in plot 1 within a 12 d time frame. The subsequent analysis of these measurements revealed notable consistency in the mean *F*_CH4_ across the three time replicates (see Table 1), displaying a CV of 13.9%. By contrast, the CV among the mean *F*_CH4_ values from the five plots was considerably higher (60.7%). Heatmaps further substantiated this similarity, illustrating comparable emission patterns among the three replicates (Figure 1B). To distinguish between temporal and spatial replicates more accurately, we compared the interpolated matrices obtained from plot 1 on the three dates. We first calculated the mean CV of *F*_CH4_ among time replicates for each of the 2500 *X*, *Y* coordinates, which was 24.1 ± 14.7%. Then, we calculated the mean CV of *F*_CH4_ among the 2500 locations, separately, for each of the three dates. The latter afforded a CV of 129–174% (*n* = 3), which was significantly higher than the mean CV among the time replicates (*p* < 0.05). Therefore, our results strongly suggest that the variability of *F*_CH4_ observed within each plot was primarily associated with spatial considerations rather than temporal ones.

Regarding the factors that might explain the spatial variability, our initial hypothesis was that terrain irregularities and the corresponding ground height relative to the water table (*H;*Figure S3) influenced *F*_CH4_, a factor often suggested in the literature.^19,20,40^ We did not observe a correlation between *H* and *F*_CH4_, except in plot 3, where a clear correlation was evident, with a Spearman’s rank correlation factor (ρ) of −0.713 and −0.846 for experimental (*n* = 16) and interpolated (*n* = 2500) data, respectively (in both cases, *p* < 0.01). These factors indicate a strong to very strong negative correlation (Figure S4).^34^ To shed light on this result, we proposed that plot 3 encompassed several features that could accentuate a potential correlation between *H* and *F*_CH4_. Notably, plot 3 lacked hotspots (*A*_FCH4_^5×^ = 0.0%) and exhibited a relatively low negative area (*A*_FCH4_^<0^= 6.4%), resembling plot 5. However, unlike plot 5, where no correlation between *H* and *F*_CH4_ was observed, plot 3 presented a relatively high *H* gradient (0.45 m, compared to 0.15 m for plot 5). Consequently, we suggested that the influence of *H* on *F*_CH4_ likely exists but is concealed by other predominant factors, such as hotspots and negative fluxes, which are independent of *H* (no correlation found; data not shown). The latter was consistent with the literature, as several studies did not find that *H* was a controlling factor over *F*_CH4_ (e.g.,^14,41−43^). This, similar to our case, was attributed to the highly heterogeneous nature of peatlands and potential oxic areas below the water table and anoxic areas above it observed at very small (cm^2^) scales.^44^

**Figure 3:**
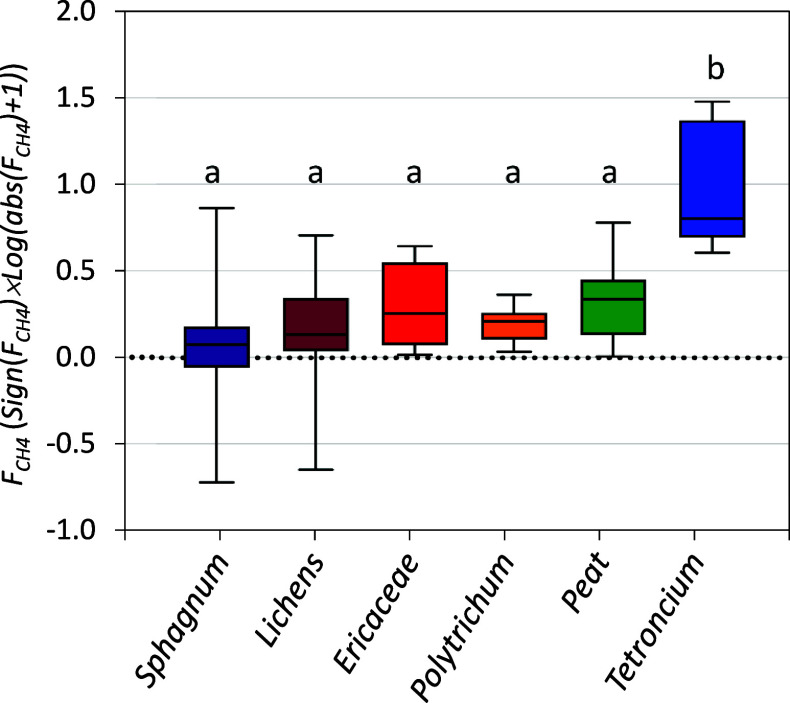
Box and whisker chart illustrating CH_4_ emissions in logarithmic scale observed for each dominant vegetational class. The vegetational class “shrubby *Nothofagus antarctica*” is not depicted due to its nondominance in any location.

In our quest to identify an explanatory factor for the substantial spatial variability in *F*_CH4_, we investigated whether vegetation coverage in nature as well as quantity significantly influences the observed *F*_CH4_ levels. We documented six plant species and two lichen species at the ground surface, occasionally observing bare peat in certain areas. Based on these observations, we categorized the vegetation coverage into seven classes: (1) *Sphagnum magellanicum* and (2) *Polytrichum strictum*, both of which are nonvascular plants, (3) *Ericaceae* species (*Empetrum rubrum* and *Gaultheria pumila*), (4) *Tetroncium magellanicum* and (5) shrubby *Nothofagus antarctica*, which are vascular plants, (6) lichens (*Cladonia arbuscula* and *Coelopogon epiphorellus*), and (7) bare peat. Utilizing the collected data, we calculated the SDI across the five plots (Figure S5). The parameter SDI exhibited a range between 0.000 and 0.573 across all locations, with a mean value of 0.230 ± 0.135; however, no correlation was found among CH_4_ emissions and species diversity. This variation in SDI is further illustrated by a shade plot depicted in Figure S6, which represents the relative abundance of the seven vegetation classes across 80 locations. On average, the *Ericaceae* class dominated in all the plots except Plot 5, where *P. strictum* was the most prevalent. Notably, although the shrubby *N. antarctica* was present in all of the plots, it was scarce, being observed only once or twice in each plot. The impact of vegetational cover was further evaluated by comparing *F*_CH4_ among the dominant species found at each location. Particularly, where *T. magellanicum* was dominant, the *F*_CH4_ levels were significantly higher than at any other location (*p* < 0.05; Figure 3), with a mean emission of >9.8 times than that of the other locations. Furthermore, *F*_CH4_ levels showed a linear relationship to the relative abundances of *T. magellanicum* in the five plots analyzed (*r* = 0.6272; *p* < 0.01; Figure S7). These notable results can be explained by the fact that *T. magellanicum* was the sole rhizomatous herb^45^ found in the terrain, and this species develops an extensive aerenchymatous system to improve oxygen transport from aboveground to the root tips.^46,47^ However, this plant structure also facilitates the inverse transport of CH_4_ (and other gases) from the soil to the atmosphere.^23,48−50^ Notably, the observed relationship between *T. magellanicum* dominance and *F*_CH4_ levels paves the way for using plant identification as a proxy for CH_4_ emissions, as previously suggested by Dias et al.^51^

In addressing potential correlations between environmental factors and *F*_CH4_ levels to explain significant emission variability, the present study failed to include soil temperature or heat balance among the measured parameters, which exhibits substantial variations in short spatial scales^52,53^ and plays a crucial role in driving the bioprocesses within the CH_4_ cycle. Future research on the spatial variability of CH_4_ cycling can be substantially advanced by incorporating soil temperature along with factors such as oxygen availability, water content beyond the water table depth, and the quality of peat organic matter. However, this prospective research should encompass depth profiles within the acrotelm where most biogeochemical processes occur.^54,55^

### 3.2 Ecosystem Respiration

The ecosystem respiration also varied widely over the 80 locations that were examined, with *R*_CO2_ ranging from 28.5 to 1231.0 mg m^–2^ h^–1^ with a mean of 356.9 ± 268.0 mg m^–2^ h^–1^ and a CV of 75.1%. This range matched those reported by earlier investigations performed in the peatlands of Southern Patagonia, where the recorded values varied from 8 to 667 mg m^–2^ h^–1^ using the conventional static chamber technique.^37,56^ As previously done with *F*_CH4_, a total of five *R*_CO2_ heatmaps were generated for the five plots (Figure S8A; A1–A5) and three heatmaps were elaborated for plot 1 corresponding to three distinct dates to account for temporal variations (Figure S8B; B1–B3). Regarding the spatial variability of *R*_CO2_, the results obtained were markedly different from that of *F*_CH4_. No hotspots or negative area (*A*_RCO2_^5×^ = 0%; *A*_RCO2_^<0^= 0%) were observed, and the CV of *R*_CO2_ within each plot was relatively small (50.4% ± 7.2% on average). All the parameters of the NHM indicated a lower heterogeneity than those of *F*_*CH4*_ (*p* < 0.05; Table 2 and Figure 2B). Moreover, in contrast to *F*_CH4_, no clear distinction between temporal and spatial variations could be made. First, the CV among the time replicates (plot 1) of the mean *R*_CO2_ (42.2% ± 4.3%) was not significantly different from the CV of the mean *R*_CO2_ among the five plots (50.4 ± 7.18%; Table 2). Second, when comparing the interpolated matrices obtained from plot 1, the mean CV of *R*_CO2_ among the time replicates (same *X* and *Y* coordinates over three dates) and among the locations (all *X* and *Y* coordinates on the same date) were similar, at 41.0 and 42.2%, respectively. Thus, distinguishing between spatial and temporal variabilities is challenging owing to their complex interplay and our data set does not provide conclusive evidence.

**Table 2:** Mean Indicators of CO_2_ Emissions and Spatiotemporal Attributes^a^

	mean *R*_CO2_ (mg m^–2^ h^–1^)	CV *R*_CO2_ (%)	*A*_RCO2_^5×^ (%)	*A*_RCO2_^<0^*(*%)	*A*_RCO2_^90^ (%)	*ht*_RCO2_ (%)
plot 1 (*t* = 0)	337.0	47.2	0.0	0.0	75.2	27.2
plot 1 (*t* = 2 d)	257.8	40.1	0.0	0.0	79.0	22.8
plot 1 (*t* = 12 d)	319.3	39.3	0.0	0.7	76.1	21.9
mean plot 1	304.7	42.2	0.0	0.2	76.8	23.9
plot 2	339.0	50.9	0.0	0.0	78.3	27.1
plot 3	436.0	53.9	0.0	0.0	72.2	30.7
plot 4	466.7	44.9	0.0	0.0	80.5	24.3
plot 5	304.6	60.2	0.0	0.0	72.6	33.9
mean 5 plots	370.2	50.4	0.0	0.0	76.1	28.0
CV 5 plots (%)	20.6	14.2	0.0	223.6	4.7	15.2

a CV, coefficient of variation; *A*_RCO2_^5×^, hotspot area; *A*_RCO2_^<0^, negative area; *A*_RCO2_^90^, area that contribute 90% of total emissions; *ht*_RCO2_, heterogeneity parameter (see text for details).

### 3.3 Discussion on the Spatial Variability

Herein, we focused on assessing the fine-scale spatial variation of *F*_CH4_ across five distinct plots. To quantify the scale of spatial variation, i.e., the minimum distance or spatial domain over which notable changes in the measured parameter occur, we calculated the relative differences in *F*_CH4_ for all of the possible pairs of locations within each plot and the distances between these two locations, resulting in a total of 120 combinations. Then, we used Spearman’s rank correlation to explore any potential relationship between the computed differences in *F*_CH4_ and their respective distances. The analysis revealed correlations ranging from −0.042 to 0.162, with a mean correlation of 0.081. The correlations were nonsignificant (*p* > 0.05) in all cases except one (plot 2, *p* = 0.048). These results suggest a weak and inconsistent monotonic relationship between the relative differences in *F*_CH4_ and the distances between the measurements. A similar approach was used through the determination of semivariograms,^57^ showing no tendency and no range, i.e., the distance beyond which the spatial autocorrelation between points becomes negligible or very low. Essentially, the latter means that the scale of spatial variation for *F*_CH4_ appears to be smaller than the distances between the measurements examined in this study. As the mean distance between contiguous measurements, i.e., the minimum tested distances, was 0.871 ± 0.248 m during our campaign (*n* = 24 for each plot), we proposed that the scale of spatial variation is smaller than this distance. Furthermore, each measurement in our campaign represented an area of 0.563 m^2^, derived by dividing 9 m^2^ (plot surface) into 16 measurements, suggesting that the scale of spatial variation was smaller than this area. Regarding the scale of the spatial variation of *R*_CO2_, the same exercise as with *F*_CH4_ could not be made, as we could not distinguish spatial and temporal variations (Section 3.2), thus making any specific spatial variation analysis unfeasible.

Regarding the impact of spatial variability on the sampling strategy, we assessed the effect of sample size (2 ≤ *n* ≤ 100) on the SEM through bootstrapping. The results are presented in Figure S9, indicating a significant difference between the determinations of *F*_CH4_ and *R*_CO2_, as anticipated, due to the lower variability of *R*_CO2_. Additionally, Figure S9 shows that for a given *n*, the SEM of *R*_CO2_ will be 3.5 times lower than that of *F*_CH4_, suggesting that determining *F*_CH4_ would require a significantly larger experimental effort than determining *R*_CO2_ to reach a similar accuracy level.

Thus, our study thoroughly explored the intricate spatial dynamics of CH_4_ and CO_2_ emissions within a subantarctic *Sphagnum* peatland. Notably, we observed a more heterogeneous distribution of *F*_CH4_ compared with that of *R*_CO2_. Our investigation revealed that the spatial scale of variation is <0.87 or 0.56 m^2^, which is, to the best of our knowledge, the shortest spatial scale tested to date and suggests that spatial variation occurs at an even smaller scale. In exploring factors influencing spatial *F*_CH4_ variation, we identified a minor effect of ground height relative to the water table, overshadowed by predominant factors such as hotspots and negative fluxes that did not correlate with the water table. Conversely, we found that the dominance of a specific species, *T. magellanicum*, significantly promoted *F*_CH4_, reaching values several times higher than those observed with other vegetation coverage. Moreover, our findings reaffirm previous knowledge regarding spatial variation and the influence of factors such as the water table and species coverage but at an unprecedentedly finer spatial scale. However, further research is necessary to better understand the factors influencing emissions on such a small spatial scale. In this quest, the skirt chamber can be a valuable tool, enabling the rapid determination of *F*_CH4_ at numerous locations and thus achieving a high spatial resolution. This can help reduce uncertainty caused by more sophisticated methods that offer detailed temporal resolution but are limited to the locations they cover within a given experimental effort. Combining these complementary methods would offer a holistic approach, encompassing detailed temporal resolution and broad spatial coverage, providing a comprehensive understanding of the dynamics in multiple dimensions.

## Supporting Information Available

Description of the chamber design and methods used for CH_4_ and CO_2_ flux measurement, including a schematic figure and a photograph (Figure S1), detailed figure describing the numerical homogeneity model (NHM; Figure S2), heatmaps of the measured ground height relative to the water table at the five plots (Figure S3), Spearman’s rank correlation between CH_4_ flux and the height relative to the water table in plot 3 (Figure S4), heatmaps of the Shannon diversity index (SDI) of the plant species found in the terrain (Figure S5), shade plot of the relative abundance of the seven vegetation classes across 80 locations (Figure S6), Pearson correlation of methane fluxes and the relative abundance of *T. magellanicum* in the five plots (Figure S7), heatmaps of CO_2_ emissions under dark conditions (Figure S8), and standard error of the mean as a function of the number of measurements of CH_4_ flux and CO_2_ respiration (Figure S9) (PDF)
