# Supplementary material for: Fine-Scale Spatial Variability of Greenhouse Gas Emissions From a Subantarctic Peatland Bog
Source: Environ Sci Technol. 2024 Apr 16;58(17):7393–402. doi: 10.1021/acs.est.3c10746 (PMC11064220; doi:10.1021/acs.est.3c10746)
Supplement: Supplementary file 1 — es3c10746_si_001.pdf [file es3c10746_si_001.pdf]

Fine-scale spatial variability of greenhouse gas emissions from a subantarctic peatland bog

Brenda Riquelme del Río[1,2], Armando Sepulveda-Jauregui[3,4], Julio A. Salas-Rabaza[1], Roy Mackenzie[1,2], Frederic Thalasso[1,5,*]

[1] Cape Horn International Center, Universidad de Magallanes, Teniente Muñoz 166, Puerto Williams, Chile.
[2] Millennium Institute Biodiversity of Antarctic and Subantarctic Ecosystems (BASE), Las Palmeras, 3425, Santiago 7800003, Chile.
[3] Environmental Biogeochemistry Laboratory, Centro de Investigación Gaia Antártica (CIGA), Universidad de Magallanes, Av. Bulnes 01855, Punta Arenas 6210427, Chile.
[4] Ecosystem Processes, Plankton and Microbial Ecology, IGB Leibniz-Institute of Freshwater Ecology and Inland Fisheries, Zur alten Fischerhütte 2, 16775 Stechlin, Germany.
[5] Departamento de Biotecnología y Bioingeniería, Centro de Investigación y de Estudios Avanzados del Instituto Politécnico Nacional (Cinvestav), Av. IPN 2508, Mexico City 07360, Mexico.

* Corresponding author phone: +52 (55) 57473320; email: thalasso@cinvestav.mx

Supporting Information

This document comprises of 14 pages, along with a cover, 3 supporting technical descriptions (SI1 – SI3), 9 supporting figures (Figure S1 – Figure S9) and 1 page with supporting references.

S1. Chamber design

As previously described by Thalasso et al. (2023) and depicted in Figure S1, the chamber used in this study was a pyramidal trunk basket (Model 47970, Spectrum, Mexico) with a base (opening) measuring 0.32 × 0.29 m and a height of 0.22 m. A low-density polyethylene film (1.4 × 1.4 m; 0.025 mm thick; Frost King, Mexico) was positioned above the chamber and securely attached to the bottom. A battery-operated fan (Portable Fan, Cazokasi, Mexico) was affixed to one of the lateral faces of the chamber, opposite the side facing the sun, and operated at an airflow speed of about 1.2 m s^{-1}. Two units of flexible polyurethane tubing (external diameter: 6 mm, internal diameter: 4 mm; PUN-6X1-DUO-BS, Festo, Mexico) were attached inside the chamber, to opposite sides of the basket, approximately two-thirds of the chamber's height, passing from below the chamber's edge and connecting to the UGGA. During flux measurements, the chamber was positioned with the opening facing downwards, the plastic skirt was expanded around the chamber, and the steel chain (0.27 kg m^{-1}) was placed above the plastic film, wrapping the base of the chamber three times (Figure S1).

S2. Flux measurements

The flux determination protocol involved four steps: step 1 – the ground air concentration (C_L) of CH_4 ($C_{L,CH4}$) and CO_2 ($C_{L,CO2}$) was measured for 5 min, just above the vegetation cover (where the chamber was placed); step 2 – the chamber was positioned and the gas concentration inside the chamber was measured. Once steady state was reached, C_B of CH_4 ($C_{B,CH4}$) was measured over a 5-minute period; step 3 – a pulse of about 1 mL of standard CH_4 (99.99 %, Linde, Chile) was injected with a plastic syringe through a septum connected on the waste line of the UGGA (returning to the chamber). It's important to note that the exact amount of CH_4 injected does not require precision, as outlined in Thalasso et al. (2023). Thus, we used small, easily transportable vials as the CH_4 source. This injection caused an abrupt and artificial increase of the CH_4 concentration, which then decreased asymptotically due to gas exchange between the chamber and the environment, i.e. the leaks that are measured with this method. The decreasing CH_4 concentration was used to determine θ_C (Ström et al. 2003). This step was maintained for 5 to 7 minutes, until a stable CH_4 concentration was observed; step 4 – a dark screen was placed on top of the chamber for 5 minutes to measure the CO_2 concentration ($C_{B,CO2}$), which correspond to the CO_2 flux in absence of light (respiration). In the present work two fluxes were measured, the CH_4 flux (F_{CH4}), and the CO_2 flux under dark conditions, thus corresponding to the CO_2 respiration rate of the ecosystem (R_{CO2}).

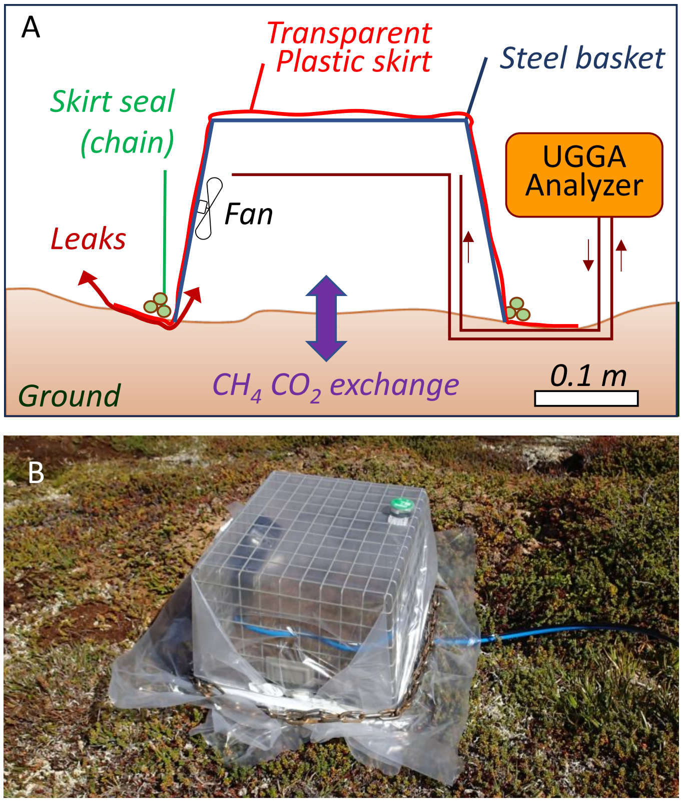

Figure S1: Skirt-chamber concept (A) and on-site photograph (B).

S3. Ebullitive versus diffusive fluxes

It is worth noting that, for its design, the skirt-chamber captures and measures the total flux reaching the chamber, including ebullitive events, well described in peatlands (Baird et al., 2019; Strack et al., 2005). In this context, the open dynamic nature of the skirt-chamber should allow distinguishing both emission modes; i.e., ebullitive and diffusive, as previously observed in a similar chamber concept deployed in freshwater ecosystems (Gerardo-Nieto et al., 2019). However, with the skirt-chamber in peatlands, we have never observed peak increases in CH_4 or CO_2 concentration that would be the result of a bubble reaching the chamber. Our hypothesis is that the skirt-chamber, deployed under non-submerged conditions with the water table depth typically ranging from 0.1 to 0.6 meters, allowed bubbles released at the water surface to undergo progressive dilution until they reached the peat surface and the chamber. This process minimizes the abrupt increase in concentration observed in chambers deployed under submerged conditions when bubbles directly reach the chamber volume.

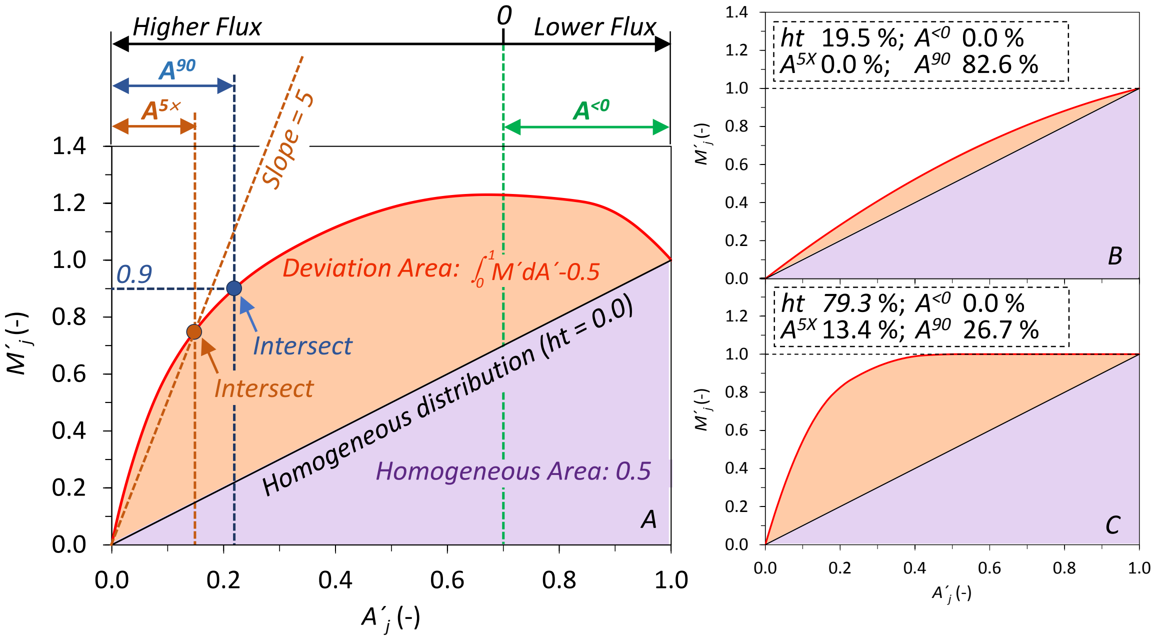

Figure S2: Graphical representation of the numerical homogeneity model (NHM). The red continuous line depicts the pair formed by the normalized cumulated and ordered mass of CH_4 emitted by the plot (M') and the corresponding normalized cumulated area (A'; Eqs. 3, 4); the purple area corresponds to the area that would be observed under a perfectly homogeneous distribution of F_{CH4}, the orange area represents the area corresponding to the deviation to that perfect case, which define the *ht* parameter (Eq. 5); $A^{<0}$ represents the percentage of area initial where negative fluxes are observed; $A^{5\times}$ indicates the percentage or area where an emission superior to five time the mean emission (hotspots) and A^{90} represents the percentage of area responsible for 90% of the total emission (see main document for details). Panel A, shows an example, based on F_{CH4} measured at Plot 4, with large heterogeneities, hotspots and negative fluxes; Panel B shows a theoretical example with small heterogeneities, no hotspots and no negative fluxes (M'_j never exceed 1.0); Panel C shows a theoretical example with large heterogeneities, hotspots and no negative fluxes.

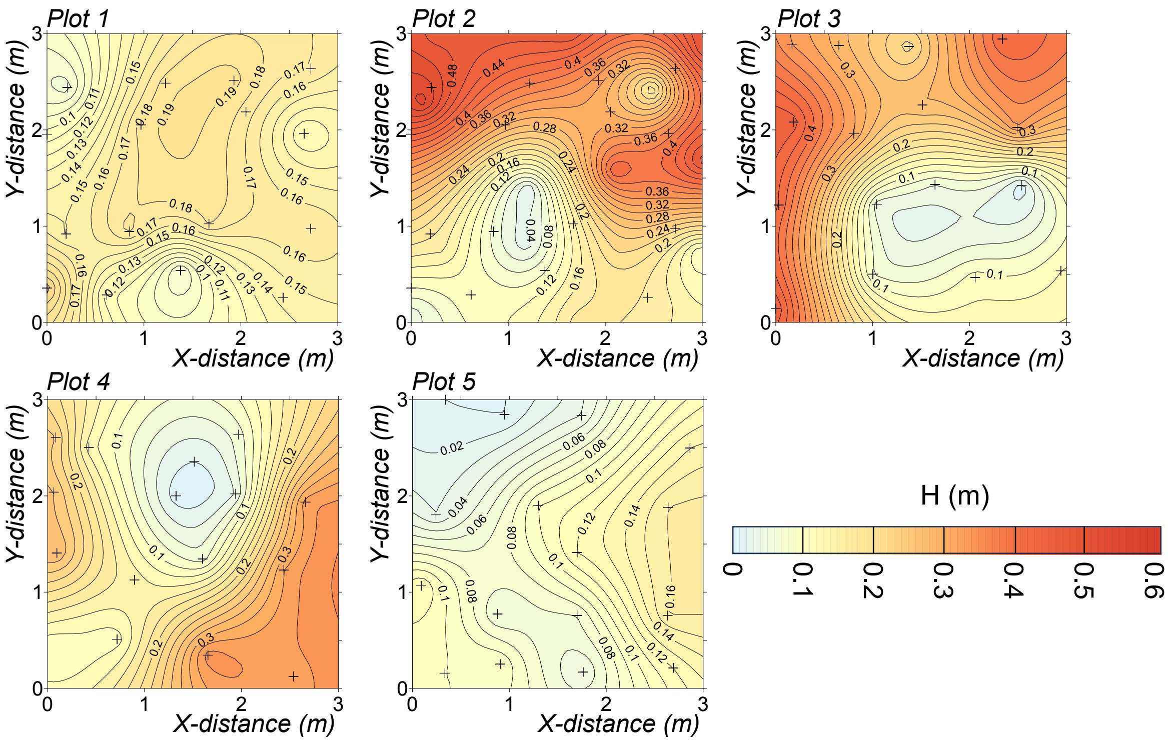

Figure S3: Heatmaps of the measured ground height relative to the water table (*H*) at the five 3 × 3 plots. Crosses indicate location where F_{CH4} were measured.

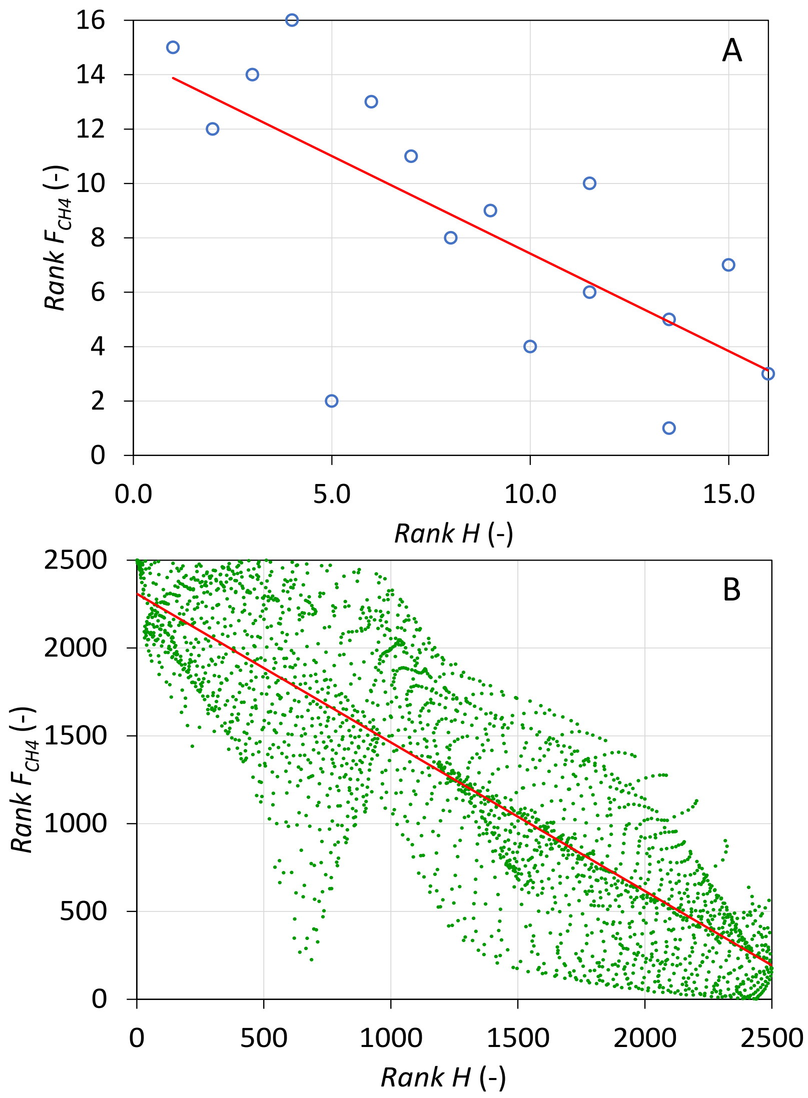

Figure S4: Spearman's rank correlation between the ground height relative to the water table (*H*), and F_{CH4} at Plot 3; (A) experimental data (ρ = -0.713; $p < 0.01$), (B) interpolation matrix data (ρ = -0.846; $p < 0.01$).

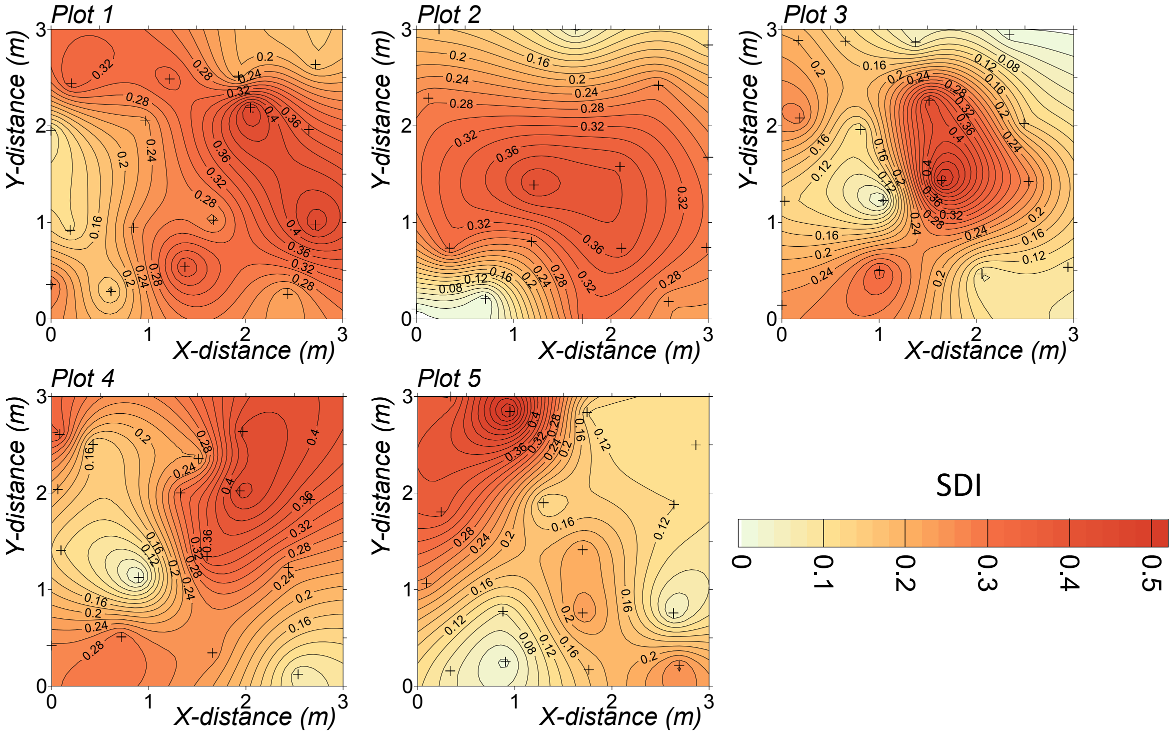

Figure S5: Heatmaps of the Shannon diversity index (*SDI*) at the five 3 × 3 plots. Crosses indicate coordinates of the measurements.

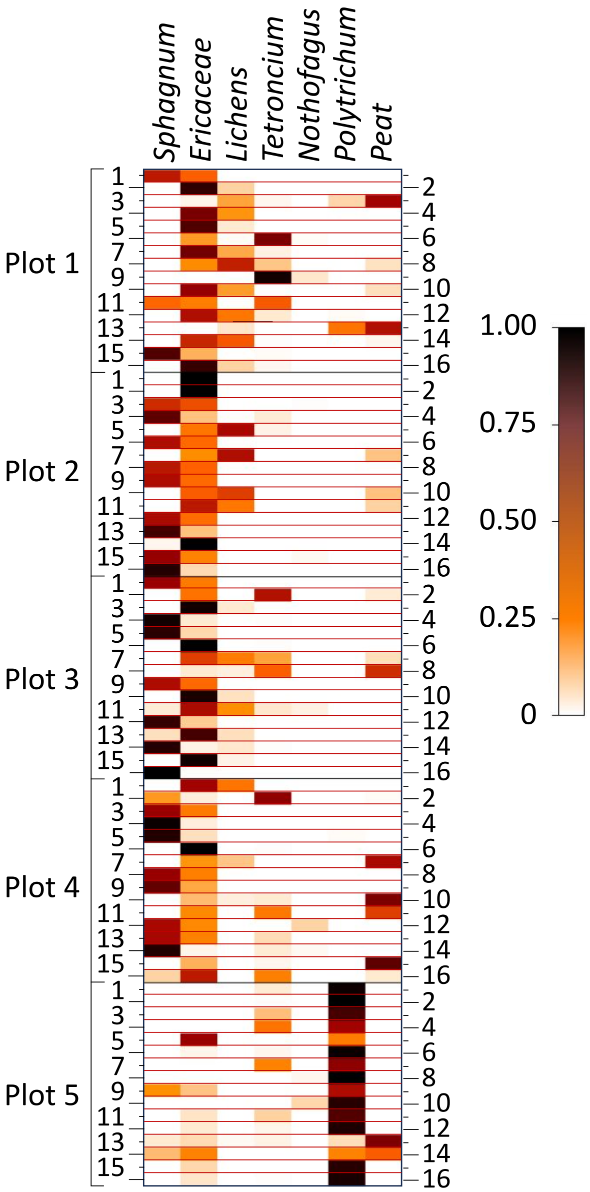

Figure S6: Shade-plot of the relative abundance of the seven vegetation classes across the 80 locations.

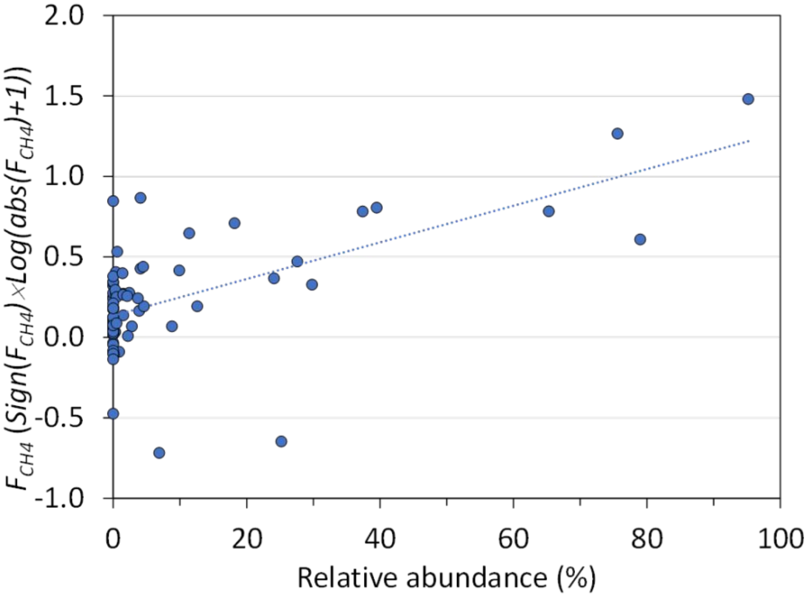

Figure S7: Methane fluxes as a function of the relative abundance of *Tetroncium magellanicum* in the 5 plots (Pearson correlation (r) = 0.6272; $p < 0.01$).

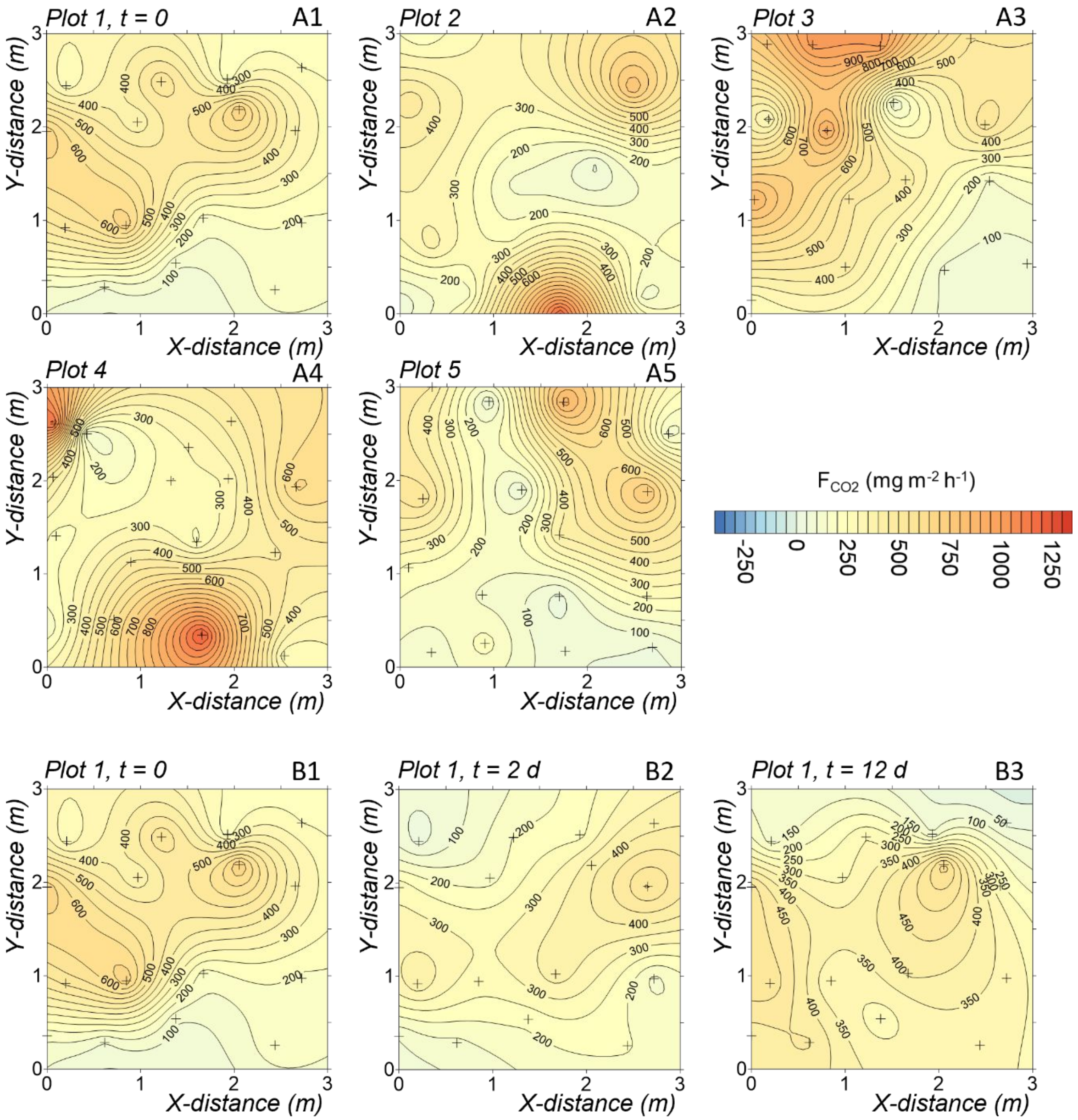

Figure S8: CO_2 emission heatmaps (R_{CO2}) over five distinct 3 × 3 plots (A1–5) and over three dates (B1–3; day 0, 2 and 12) on the same plot as A1. The color scale is linear. Crosses indicate coordinates of the measurements.

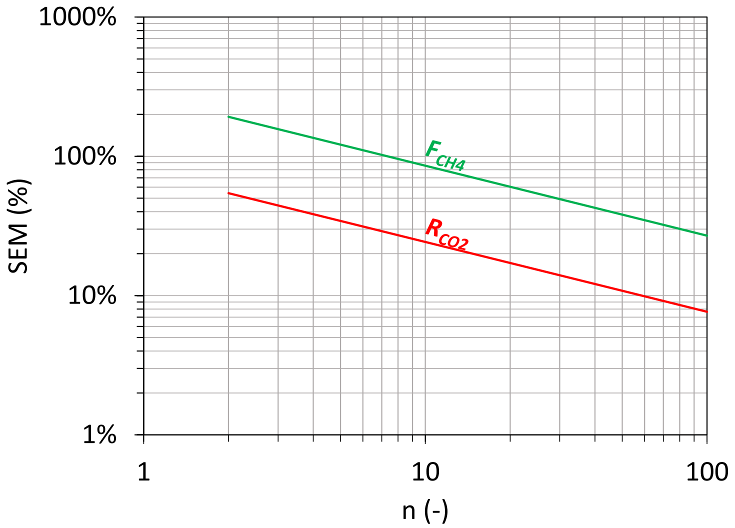

Figure S9: Standard Error of the Mean (%) as a function of the number of measurements of F_{CH4} and R_{CO2}, determined by bootstrapping (1000 iterations).

Supporting references

Baird, A. J.; Green, S. M.; Brown, E.; Dooling, G. P. Modelling time-integrated fluxes of CO_2 and CH_4 in peatlands: A review. *Mires Peat* **2019**, *24*, 1–15.

Ström, L.; Ekberg, A.; Mastepanov, M.; Christensen, T. R. The effect of vascular plants on carbon turnover and methane emissions from a tundra wetland. *Glob. Chang. Biol.* **2003**, *9*, 1185–1192.

Thalasso, F.; Riquelme, B.; Gómez, A.; Mackenzie, R.; Aguirre, F. J.; Hoyos-Santillan, J.; Rozzi, R.; Sepulveda-Jauregui, A.. Technical note: Skirt chamber – an open dynamic method for the rapid and minimally intrusive measurement of greenhouse gas emissions from peatlands. *Biogeosciences* **2023**, *20*, 3737–3749.

Strack, M.; Kellner, E.; Waddington, J. M. Dynamics of biogenic gas bubbles in peat and their effects on peatland biogeochemistry. *Glob. Biogeochem. Cycles*, **2005**, *19*, GB1003. https://doi.org/10.1029/2004GB002330.

Gerardo-Nieto, O.; Vega-Peñaranda, A.; González-Valencia, R.; Alfano-Ojeda, Y.; Thalasso, F. Continuous measurement of diffusive fluxes of methane in aquatic ecosystems by an open Dynamos chamber method. *Environ. Sci. Technol.*, **2019**, *53* (9), 5159-5167.
